# Prognostic factors in patients with hepatitis B virus-related hepatocellular carcinoma undergoing nucleoside analog antiviral therapy

**DOI:** 10.3892/ol.2013.1578

**Published:** 2013-09-12

**Authors:** HIROKI NISHIKAWA, NORIHIRO NISHIJIMA, AKIRA ARIMOTO, TADASHI INUZUKA, RYUICHI KITA, TORU KIMURA, YUKIO OSAKI

**Affiliations:** 1 Department of Gastroenterology and Hepatology, Osaka Red Cross Hospital, Osaka 543-0027, Japan; 2 Department of Surgery, Osaka Red Cross Hospital, Osaka 543-0027, Japan; 3 Departments of Gastroenterology and Hepatology Surgery, Graduate School of Medicine, Kyoto University, Kyoto 606-8501, Japan

**Keywords:** hepatitis B virus, hepatocellular carcinoma, entecavir, prognostic factor

## Abstract

In the present era of entecavir (ETV) use for chronic hepatitis B (CHB), the prognostic factors in hepatitis B virus (HBV)-related hepatocellular carcinoma (HCC) remain unclear. The aims of the present study were to investigate the prognostic factors in patients with HBV-related HCC treated with ETV who underwent curative therapy. A total of 74 HBV-related HCC patients treated with ETV who underwent curative therapy were analyzed. Predictive factors associated with overall survival (OS) and recurrence-free survival (RFS) were examined using univariate and multivariate analysis. Our study population included 49 males and 25 females with a median age of 62 years. The median observation period was 3.4 years (range, 0.2–11.5 years). The 1-, 3- and 5-year cumulative OS rates were 100, 89.8 and 89.8%, respectively. The corresponding RFS rates were 82.8, 52.1 and 25.6%, respectively. In this study, 73 patients (98.6%) achieved an HBV DNA level of <400 copies/ml during the follow-up period. No viral breakthrough hepatitis, as defined by 1 log increase from nadir, was observed during ETV therapy. According to multivariate analysis, only hepatitis B e antigen (HBeAg) positivity was significantly associated with OS [hazard ratio (HR), 0.058; 95% confidence interval (CI), 0.005–0.645; P=0.020)], whereas HCC stage (HR, 0.359; 95% CI, 0.150–0.859; P=0.021), HBeAg positivity (HR, 0.202; 95% CI, 0.088–0.463; P<0.001) and γ-glutamyl transpeptidase ≥50 IU/l (HR, 0.340; 95% CI, 0.152–0.760; P=0.009) were significant predictive factors linked to RFS. In conclusion, HBeAg positivity was significantly associated with OS and RFS in HBV-related HCC patients treated with ETV who underwent curative therapy. In such patients, close observation is required, even after curative therapy for HCC.

## Introduction

Hepatocellular carcinoma (HCC) is a major health problem worldwide. It is the fifth most common type of cancer in males and the seventh most common in females, as well as the third most common cause of cancer-related mortality (1–3). Chronic hepatitis B (CHB) is the leading cause of HCC development in Asia, although Japan has one of the lowest prevalence rates for CHB among Asian countries (4). Each year, more than 50 million people are infected with HBV worldwide and more than 1 million deaths are attributed to HBV-related complications, including liver cirrhosis and HCC (5,6).

In the majority of HCC patients, successful treatment of HCC is followed by recurrence, leading to high mortality rates (7). Thus, the prediction of HCC recurrence and the performance of appropriate therapy for HCC recurrence after initial treatment are essential for the optimization of clinical outcomes (8).

Lamivudine (LAM) was the first nucleoside analog (NA) introduced for the treatment of CHB. In several clinical trials, it showed superior efficacy compared with the placebo in terms of HBV DNA suppression, hepatitis B e antigen (HBeAg) seroconversion and alanine aminotransferase (ALT) normalization (9,10). However, a major limitation of LAM therapy is the development of resistance, which occurs in up to 70% of patients within 4 years of therapy (11). Adefovir (ADV) is not cross-resistant with LAM and has been used for the treatment of CHB. However, in two pivotal phase III clinical trials of ADV for patients with CHB, among subjects who received a 10-mg dose once daily, only 21% of HBeAg-positive patients and 51% of HBeAg-negative patients achieved a serum HBV DNA level of <400 copies/ml at 48 weeks (12,13).

Entecavir (ETV) is a cyclopentyl guanosine analog that has demonstrated superior virological, biochemical and histological effects as compared with those of LAM and ADV in large randomized controlled trials, and is now widely used as a first choice NA with the purpose of improving clinical outcome in CHB patients (14–18). For LAM-treated patients with no viral breakthrough, switching therapy to ETV is also recommended (19). In addition, it has been shown recently that ETV is able to reduce the risk of HCC occurrence and liver-related mortality in CHB patients (20,21). However, to the best of our knowledge, predictive factors in HBV-related HCC patients treated with ETV who have undergone curative therapy remain unclear, and it is essential for clinicians to examine these factors to optimize their clinical outcomes. Therefore, the aims of the present study were to elucidate the prognostic factors in patients with HBV-related HCC treated with ETV who underwent curative therapy.

## Patients and methods

### Patients

A total of 131 treatment-naïve HBV-related HCC patients received curative therapy at Osaka Red Cross Hospital (Osaka, Japan) between January 2001 and November 2012. They were all positive for HB surface antigen (HBsAg) and negative for anti-HCV (HCV Ab). Curative therapy was defined as therapy resulting in no apparent viable tumor on a dynamic computed tomography (CT) performed within one month after initial treatment for HCC. Following diagnosis of HCC, the most appropriate therapeutic procedure was selected after discussions with surgeons and physicians, according to the tumor characteristics and underlying liver functional reserve of each patient. Of the aforementioned 131 treatment-naïve HBV-related HCC patients, 32 did not receive NA therapy, 69 received ETV monotherapy, 18 received ADV add-on treatment having converted from LAM monotherapy due to breakthrough hepatitis, 5 received ETV monotherapy having switched from LAM monotherapy and 7 received LAM monotherapy (19,22,23). Thus, a total of 74 HBV-related HCC patients treated with ETV were analyzed in the present study. Predictive factors linked to overall survival (OS) and recurrence-free survival (RFS) rates were examined.

Written informed consent was obtained from all patients prior to each therapy, and the study protocol complied with all the provisions of the Declaration of Helsinki. This study was approved by the Ethics Committee of Osaka Red Cross Hospital, Japan, and the need for written informed consent was waived as the data were analyzed retrospectively and anonymously. The present study comprised a retrospective analysis of patient records registered in our database, and all treatments were conducted in an open-label manner.

### HCC and liver cirrhosis (LC) diagnosis

HCC was diagnosed using abdominal ultrasound and dynamic CT scans (hyperattenuation during the arterial phase in all or some part of the tumor and hypoattenuation in the portal-venous phase), and/or magnetic resonance imaging (MRI), mainly based on the recommendations of the American Association for the Study of Liver Diseases (24). Arterial- and portal-phase dynamic CT images were obtained at ~30 and 120 sec, respectively, after the injection of the contrast material. HCC stage was determined using the Liver Cancer Study Group of Japan staging system (25). HCC was confirmed pathologically only in patients who underwent surgery. LC was determined by specimens at surgery, imaging modalities or portal hypertension, such as esophageal varices and splenomegaly.

### Serological studies

HBsAg, HCV Ab, HBeAg and HBeAb were detected using commercial enzyme immunoassay kits (Architect, Dainabot, Tokyo, Japan; Lumipulse; Fujirebio Inc, Tokyo, Japan). HBV DNA levels were quantified using the COBAS^®^ Amplicor HBV Monitor Test (Roche Diagnostics, Tokyo, Japan), which has a dynamic range of 2.6–7.6 log copies/ml, or the COBAS TaqMan^®^ HBV Test (version 2.0; Roche Diagnostics), which has a dynamic range of over 2.1–9.0 log copies/ml.

### Follow-up

Follow-up after each therapy consisted of periodic blood tests and monitoring of tumor markers, including α-fetoprotein (AFP) and des-γ-carboxy prothrombin (DCP), using chemiluminescent enzyme immunoassays (Lumipulse PIVKA-II Eisai; Eisai, Inc., Tokyo, Japan). Dynamic CT scans and/or MRI were obtained every 2–4 months after each therapy. Chest CT, whole abdominal CT, brain MRI and bone scintigraphy were performed when extrahepatic HCC recurrence was suspected.

### Statistical analysis

Data were analyzed using univariate and multivariate analyses. Time to recurrence was defined as the interval between each therapy and the first confirmed recurrence. For analysis of RFS, follow-up ended at the time of first recurrence; other patients were censored at their last follow-up visit or at the time of death from any cause without recurrence. For analysis of OS, follow-up ended at the time of death from any cause, and the remaining patients were censored at the last follow-up visit. The cumulative OS and RFS rates were calculated using the Kaplan-Meier method and tested using the log-rank test. Factors with P<0.2 in the univariate analysis were subjected to multivariate analysis using the Cox proportional hazards model. These statistical methods were used to estimate the interval from the initial treatment for HCC. Data were analyzed using SPSS software (SPSS Inc., Chicago, IL, USA) for Microsoft Windows. Data are expressed as the mean ± standard deviation. Values of P<0.05 were considered to indicate a statistically significant difference.

## Results

### Baseline characteristics

The baseline characteristics of the patients at initial treatment for HBV-related HCC in the present study (n=74) are shown in Table I. Patients included 49 males and 25 females with a median age of 62 years. The median observation period was 3.4 years (range, 0.2–11.5 years). Surgical resection was performed in 30 patients (40.5%), and percutaneous ablation therapy such as radiofrequency ablation (RFA) and percutaneous ethanol injection (PEI) was performed in 44 patients (59.5%). Treatment procedure-related mortality was not observed in any of the patients. NAs were being administered to 47 patients at the time of initial treatment for HCC, while the remaining 27 patients had received NA therapy prior to initial treatment. Thirty-five patients (47.3%) had a pre-treatment HBV DNA level of >10^5^ copies/ml and 20 patients (27.0%) had HBeAg positivity at initial treatment for HCC.

### Cumulative OS and RFS rates

**Figure 1:**
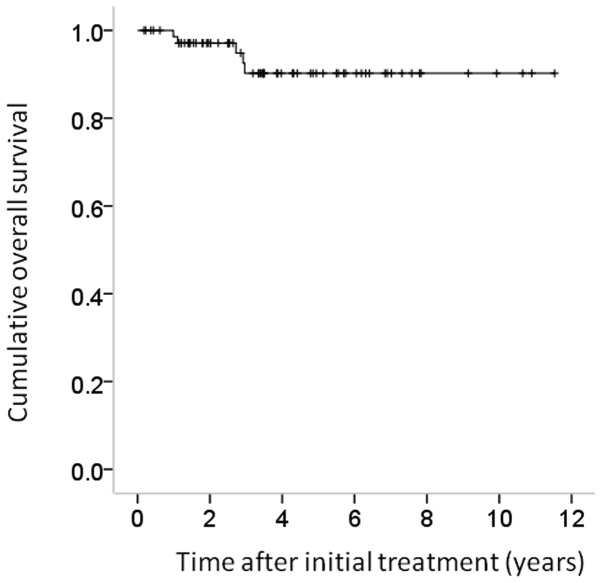
Cumulative overall survival (OS) following initial treatment for hepatocellular carcinoma for all cases (n=74). The 1-, 3- and 5-year cumulative OS rates were 100, 89.8 and 89.8%, respectively.

**Figure 2:**
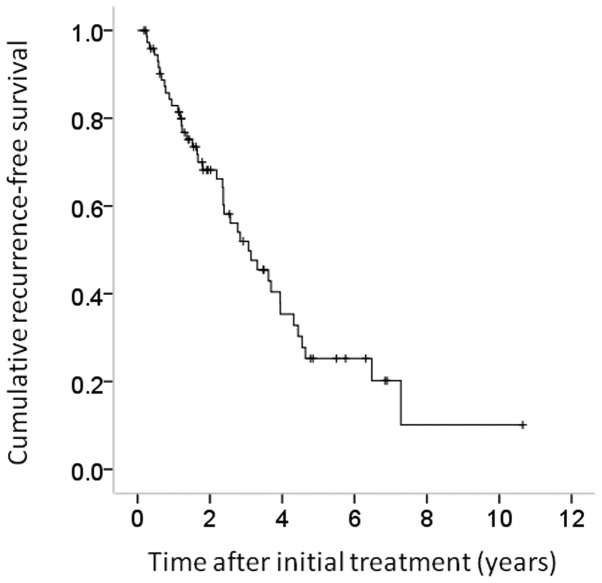
Cumulative recurrence-free survival (RFS) following initial treatment for hepatocellular carcinoma for all cases (n=74). The 1-, 3- and 5-year cumulative RFS rates were 82.8, 52.1 and 25.6%, respectively.

The 1-, 3- and 5-year cumulative OS rates for all cases were 100, 89.8 and 89.8%, respectively (Fig. 1). The corresponding RFS rates for all cases were 82.8, 52.1 and 25.6%, respectively (Fig. 2).

### Univariate and multivariate analyses of factors contributing to OS

Univariate analysis identified HbeAg positivity (P=0.003) as the only factor significantly associated with OS for all cases (n=74) (Table II). The hazard ratios (HRs) and 95% CIs calculated using multivariate analysis for the five factors with P<0.2 in the univariate analysis are detailed in Table II. Only HBeAg positivity (P=0.020) was revealed to be a significant predictor of OS in the multivariate analysis.

### Univariate and multivariate analyses of factors contributing to RFS

Univariate analysis identified the following factors as significantly associated with RFS for all cases (n=74): Presence of LC (P=0.017), HBeAg positivity (P<0.001), serum albumin ≥4.2 g/dl (P=0.003) and presence of diabetes mellitus (P=0.028) (Table III). The HRs and 95% CIs calculated using multivariate analysis for the seven factors with P<0.2 in the univariate analysis are detailed in Table III. HCC stage (P=0.021), HBeAg positivity (P<0.001) and γ-glutamyl transpeptidase (GGT) ≥50 IU/l (P=0.009) were found to be significant prognostic factors linked to RFS.

### HBeAg seroconversion, HBeAg loss and HBsAg loss

In the present study, 20 patients had HBeAg positivity at initial treatment for HCC. Of these patients, HBeAg seroconversion was observed in nine patients (45.0%) during the observation period, and HBeAg loss without HBeAg seroconversion was observed in one patient (5.0%). None of the patients experienced HBsAg loss during the observation period.

### Effect of ETV therapy on the reduction of HBV DNA viral load and ETV-related serious adverse events (SAEs)

In this study, 73 patients (98.6%) achieved an HBV DNA level of <400 copies/ml during the follow-up period. No viral breakthrough hepatitis, as defined by 1 log increase from nadir, was observed during ETV therapy. No ETV-related SAEs were observed.

### Causes of death

In the present study, five patients (6.8%) died during the follow-up period. The causes of death were HCC recurrence in four patients and miscellaneous causes in one patient.

### HCC recurrence

In the present study, 42 patients (56.8%) exhibited HCC recurrence during the follow-up period. The patterns of HCC recurrence after initial treatment were: Single HCC recurrence in the liver in 25 patients, multiple HCC recurrences in the liver in 14 patients, multiple HCC recurrences in the liver with lung metastases in one patient, multiple HCC recurrences in the liver with bone metastases in one patient and single lymph node metastasis in one patient. Treatment methods for the first HCC recurrence were: Surgical resection in three patients, percutaneous ablation therapy in 31 patients, transcatheter arterial chemoembolization in six patients and systemic chemotherapy in two patients.

## Discussion

To the best of our knowledge, there have been no studies regarding predictive factors in HBV-related HCC patients treated with ETV who have undergone curative therapy, despite the fact that ETV is now a first-line NA therapy for patients with CHB due to the superior efficacy of HBV DNA suppression, ALT normalization and histological improvement compared with LAM and ADV treatment (14–18). In the present era of NA treatment for CHB patients, the identification of predictors in HBV-related HCC patients treated with ETV is essential for improved prognosis. Hence, we conducted this retrospective analysis.

In the multivariate analysis, HBeAg positivity was the only independent predictor of OS. Although several studies have reported that liver function-related factors (such as serum albumin level) and tumor-related factors (such as HCC stage, maximum tumor size and tumor markers) are closely associated with OS in patients with HBV-related HCC, the majority of these studies did not use ETV as an antiviral therapy (26–28). In the present era of ETV use, HBV viral status rather than liver function or tumor-related factors may influence OS in patients with HBV-related HCC.

In the present study, HBeAg positivity was also significantly associated with RFS in the multivariate analysis. Sun *et al*(29) reported that HBeAg is associated with a higher risk of early recurrence and poorer survival in patients following curative resection of small HCC. The results of the present study were similar to their findings; in HBV-related HCC patients with HBeAg positivity, careful observation for HCC recurrence is required after curative therapy. Notably, serum GGT level was a significant factor contributing to RFS in the multivariate analysis in this study. Several studies have reported that a high level of GGT is related to a higher incidence of HCC development and recurrence (30,31). In HBV-related HCC patients with higher GGT levels at initial treatment, close observation for HCC recurrence is also required after curative therapy. HCC stage is also an independent predictor linked to RFS. Even in HCC patients who have undergone curative therapy, clinicians should be aware of tumor-related factors.

Resistance to NAs is a major issue affecting long-term NA therapy. However, in our results, 73 patients (98.6%) achieved an HBV DNA level of <400 copies/ml during the follow-up period, and no viral breakthrough hepatitis, as defined by 1 log increase from nadir, was observed during ETV therapy with the median follow-up period of 3.4 years. No ETV-related SAEs were identified. In addition, no patients succumbed to liver failure in the present study. Our results indicate that ETV therapy for patients with HBV-related HCC had a strong antiviral effect, maintained liver function, had a high genetic barrier to resistance and was a well-tolerated therapy as previously reported (14–16,18–21,28,32).

Higher HBV viral load was not a significant factor in terms of OS and RFS, although several studies have demonstrated that pretreatment HBV viral load was an independent predictor linked to clinical outcomes (33–35). A possible reason for this is that in the majority of patients with high HBV viral load in our study, the HBV viral load was reduced to lower HBV DNA level by ETV therapy, resulting in improved OS and RFS.

The present study had certain limitations. First, this is a retrospective study with a heterogeneous patient population. Second, the number of patients in our study was small for survival analysis. Further prospective studies with a sufficient sample size will thus be required in the future. However, the results of this study demonstrate that HBeAg positivity at initial treatment for HBV-related HCC was a significant predictor of OS and RFS following curative therapy.

In conclusion, in the present era of ETV as a first-line therapy for CHB, HBeAg positivity may be a useful predictor of survival in HBV-related HCC patients after curative therapy.

## Figures and Tables

**Table I:** Baseline characteristics at initial treatment (n=74).

Variables at initial therapy	No. or median (range)
Age (years)	62 (32–84)
Gender (male/female)	49/25
HCC stage (I/II/III)	19/40/15
Surgery/ablative therapy^a^	30/44
Maximum tumor size (cm)	2.3 (0.9–12.0)
Tumor number (single/multiple)	51/23
Liver cirrhosis (yes/no)	41/33
HBV DNA ≥ 10^5^ copies/ml (yes/no)	35/39
HBe antigen (positive/negative)	20/54
AST (IU/l)	37 (17–156)
ALT (IU/l)	31 (8–209)
ALP (IU/l)	308 (43–1446)
GGT (IU/l)	45 (11–602)
Serum albumin (g/dl)	4.1 (3.0–4.7)
Total bilirubin (mg/dl)	0.8 (0.3–4.1)
Prothrombin time (%)	84 (52–129)
Platelets (×10^4^/mm^3^ )	12.1 (1.6–63.0)
AFP (ng/ml)	18.4 (1.9–31720)
DCP (mAU/ml)	28.5 (10–102190)
Diabetes mellitus (yes/no)	8/66
Body mass index (kg/m^2^)	22.8 (16.7–36.6)

a Initial treatment for HCC.

HCC, hepatocellular carcinoma; HBV, hepatitis B virus; HBe, hepatitis B e; AST, aspartate aminotransferase; ALT, alanine aminotransferase; ALP, alkaline phosphatase; GGT, γ-glutamyl transpeptidase; AFP, α-fetoprotein; DCP, des-γ-caroxy prothrombin.

**Table II:** Univariate and multivariate analysis contributing to overall survival.

		Univariate analysis	Multivariate analysis	
			
Variables at initial treatment	No.	P-value^a^	Hazard ratio (95% CI)	P-value^b^
Gender (male vs. female)	49/25	0.572		
Age (years) (≥60 vs. <60)	43/31	0.910		
HCC stage (I or II vs. III)	59/15	0.131	0.143 (0.009–2.327)	0.172
Maximum tumor size (cm) (≥2.5 vs. <2.5)	32/42	0.927		
Tumor number (single vs. multiple)	23/51	0.096	0.777 (0.078–7.776)	0.830
Liver cirrhosis (yes vs. no)	41/33	0.295		
HBe antigen (positive vs. negative)	20/54	0.003	0.058 (0.005–0.645)	0.020
HBV DNA (≥10^5^ copies/ml vs. <10^5^ copies/ml)	35/39	0.827		
AST (IU/l) (≥40 vs. <40)	34/40	0.518		
ALT (IU/l) (≥40 vs. <40)	33/41	0.170	0.305 (0.030–3.125)	0.317
ALP (IU/l) (≥300 vs. <300)	40/34	0.795		
GGT (IU/l) (≥50 vs. <50)	35/39	0.607		
Serum albumin (g/dl) (≥4.2 vs. <4.2)	33/41	0.785		
Total bilirubin (mg/dl) (≥1.0 vs. <1.0)	27/47	0.686		
Platelet count (×10^4^/mm^3^) (≥12 vs. <12)	37/37	0.716		
Prothrombin time (%) (≥80 vs. <80)	42/32	0.387		
Serum AFP (ng/ml) (≥20 vs.<20)	37/37	0.555		
DCP (mAU/ml) (≥30 vs. <30)	36/38	0.719		
Diabetes mellitus (yes vs. no)	8/66	0.560		
Body mass index ≥23 kg/m^2^ (yes vs. no)	36/38	0.183	0.100 (0.007–1.485)	0.094

a Log-rank test;

b Cox proportional hazard model.

HCC, hepatocellular carcinoma; HBe, hepatitis B e; HBV, hepatitis B virus; AST, aspartate aminotransferase; ALT, alanine aminotransferase; ALP, alkaline phosphatase; GGT, γ-glutamyl transpeptidase; AFP, α-fetoprotein; DCP, des-γ-carboxy prothrombin; CI, confidence interval.

**Table III:** Univariate and multivariate analysis contributing to recurrence-free survival.

		Univariate analysis	Multivariate analysis	
			
Variables at initial treatment	No.	P-value^a^	Hazard ratio (95% CI)	P-value^b^
Gender (male vs. female)	49/25	0.847		
Age (years) (≥60 vs. <60)	43/31	0.598		
HCC stage (I or II vs. III)	59/15	0.154	0.359 (0.150–0.859)	0.021
Maximum tumor size (cm) (≥2.5 vs. <2.5)	32/42	0.539		
Tumor number (single vs. multiple)	23/51	0.283		
Liver cirrhosis (yes vs. no)	41/33	0.017	0.394 (0.135–1.148)	0.088
HBe antigen (positive vs. negative)	20/54	<0.001	0.202 (0.088–0.463)	<0.001
HBV DNA (≥10^5^ copies/ml vs. <10^5^ copies/ml)	35/39	0.853		
AST (IU/l) (≥40 vs. <40)	34/40	0.482		
ALT (IU/l) (≥40 vs. <40)	33/41	0.644		
ALP (IU/l) (≥300 vs. <300)	40/34	0.237		
GGT (IU/l) (≥50 vs. <50)	35/39	0.160	0.340 (0.152–0.760)	0.009
Serum albumin (g/dl) (≥4.2 vs. <4.2)	33/41	0.003	1.642 (0.712–3.787)	0.245
Total bilirubin (mg/dl) (≥1.0 vs. <1.0)	27/47	0.43		
Platelet count (×10^4^/mm^3^) (≥12 vs. <12)	37/37	0.148	0.525 (0.208–1.322)	0.172
Prothrombin time (%) (≥80 vs. <80)	42/32	0.295		
Serum AFP (ng/ml) (>≥20 vs. <20)	37/37	0.503		
DCP (mAU/ml) (≥30 vs. <30)	36/38	0.344		
Diabetes mellitus (yes vs. no)	8/66	0.028	0.987 (0.386–2.523)	0.978
Body mass index ≥23 kg/m^2^ (yes vs. no)	36/38	0.205		

a Log-rank test;

b Cox proportional hazard model.

HCC, hepatocellular carcinoma; HBe, hepatitis B e; HBV, hepatitis B virus; AST, aspartate aminotransferase; ALT, alanine aminotransferase; ALP, alkaline phosphatase; GGT, γ-glutamyl transpeptidase; AFP, α-fetoprotein; DCP, des-γ-carboxy prothrombin; CI, confidence interval.
